# Tuning the Density of Poly(ethylene glycol) Chains to Control Mammalian Cell and Bacterial Attachment

**DOI:** 10.3390/polym9080343

**Published:** 2017-08-05

**Authors:** Ahmed Al-Ani, Hitesh Pingle, Nicholas P Reynolds, Peng-Yuan Wang, Peter Kingshott

**Affiliations:** 1 Department of Chemistry and Biotechnology, School of Science, Faculty of Science, Engineering and Technology, Swinburne University of technology, Hawthorn, VIC 3122, Australia; aalani@swin.edu.au (A.A-A.); hpingle@swin.edu.au (H.P.); 2 ARC Training Centre for Biodevices, Faculty of Science, Engineering and Technology, Swinburne University of Technology, Hawthorn, VIC 3122, Australia; nreynolds@swin.edu.au

**Keywords:** PEG, surface modification, protein adsorption, cell attachment, biofilm formation

## Abstract

Surface modification of biomaterials with polymer chains has attracted great attention because of their ability to control biointerfacial interactions such as protein adsorption, cell attachment and bacterial biofilm formation. The aim of this study was to control the immobilisation of biomolecules on silicon wafers using poly(ethylene glycol)(PEG) chains by a “grafting to” technique. In particular, to control the polymer chain graft density in order to capture proteins and preserve their activity in cell culture as well as find the optimal density that would totally prevent bacterial attachment. The PEG graft density was varied by changing the polymer solubility using an increasing salt concentration. The silicon substrates were initially modified with aminopropyl-triethoxysilane (APTES), where the surface density of amine groups was optimised using different concentrations. The results showed under specific conditions, the PEG density was highest with grafting under “cloud point” conditions. The modified surfaces were characterised with X-ray photoelectron spectroscopy (XPS), ellipsometry, atomic force microscopy (AFM) and water contact angle measurements. In addition, all modified surfaces were tested with protein solutions and in cell (mesenchymal stem cells and MG63 osteoblast-like cells) and bacterial (*Pseudomonas aeruginosa*) attachment assays. Overall, the lowest protein adsorption was observed on the highest polymer graft density, bacterial adhesion was very low on all modified surfaces, and it can be seen that the attachment of mammalian cells gradually increased as the PEG grafting density decreased, reaching the maximum attachment at medium PEG densities. The results demonstrate that, at certain PEG surface coverages, mammalian cell attachment can be tuned with the potential to optimise their behaviour with controlled serum protein adsorption.

## 1. Introduction

Recently, polymer surfaces have attracted great attention because of their enormous range of applications [1,2]. Polymer brushes can be created on surfaces by either “grafting to” or “grafting from” approaches. With the “grafting to” technique, the surface is usually exposed to the desired polymer in solution or as a melt. In this case, the polymer chain gets attached to the substrate by strong interactions between one end of each chain and the substrate [3]. These interactions include charged groups [4], highly polar groups [5] and chemically reactive groups [6]. In addition, formation of tethered polymer brushes, including their generation under ambient conditions, allows precise control of polymer molecular weight, and polymer polydispersity because chains with the desired characteristics can be synthesised and purified in advance. With the “grafting from” approach, the polymerization occurs directly from the surface using a surface immobilised initiator in the presence of a monomer solution and catalyst. The graft density of polymer chains generated in this way is determined by the concentration of surface initiators and is thought to be higher than the “grafting to” approach; however, the molecular weight is regarded as being difficult to control with high polydispersities [7]. Surface polymer coatings can enhance the biocompatibility, corrosion resistance and wettability, and tailor material functionality, mechanical properties and topography for a specific application, including longer-circulating drug delivery vehicles [8]. For example, polymer grafting can be used to produce polymer brushes with specific features on the surfaces. Such brushes can contain reactive end functional groups for further covalent conjugation of active agents. Furthermore, these functional groups can give specific chemical robustness and interfacial behaviour for selective immobilisation of proteins and enzymes to the surface [9]. In addition, surface modification includes prevention of non-specific protein adsorption, which impacts the modification process. Furthermore, the architecture of the surface including polymer chain length and graft density also impact the level of protein adsorption [10]. In contrast to this, these types of surfaces are unable to minimise unwanted adhesion of bacteria and cells [11,12,13]. At present, there are a few materials that can be used to modify surfaces to reduce non-specific adsorption of proteins, particularly poly(ethylene glycol) (PEG) [14], *N*-isopropylacrylamoide (NIPAAm) [15], poly(2-ethyl-2-oxazoline) [16], zwitterionic polymers [13] and natural polymers such as dextran [17] and pullulan [18]. On the other hand, other modification techniques comprised of methods to capture specific proteins or enzymes on a surface are aimed at preserving their activity without any denaturation [19].

Despite this, non-specific protein adsorption and denaturation on the polymer brushes remains unclear. In the past, researchers found that protein adsorption decreases with increasing grafting density of PEG, which is attributed to an increase in PEG hydration and steric repulsion that reaches a maximum at the highest chain density [20,21,22,23,24]. However, this hypothesis neglects the effect of grafting density on the conformational change of protein after adsorption to lower density chains. On the other hand, the rate of protein adsorption decreases at a high polymer density of PEG, and this leads to increases in adsorbed proteins without any denaturation, which leads to an increase in residence time of proteins at the surface. Therefore, increasing the total adsorption of proteins might be a result of differences in the surface residence time of folded and un-folded protein molecules, instead of an increase in the overall amount of adsorbed proteins. In addition, higher unfolding of proteins at high polymer density may cause surface aggregation, and this may lead to further increases in the residence time of protein molecules on the polymer brush. In total, this limitation excludes the use of PEG chains for improved biocompatibility and then limits the use of PEG as a coating to resist protein adsorption. Therefore, to clarify the relationship between protein and PEG density, it is important to take into consideration protein adsorption and conformation on the PEG chains [25]. In this study, we tested the adsorption of proteins, mammalian cell and bacterial attachment on PEG chains of different grafting density in a systematic way to find an optimal surface modification protocol that could be of benefit for the applications of PEGylated biomaterials. We do that by employing a “grafting to” approach that is able to change the surface density of PEG chains by exploiting both the variations in solubility during grafting and by changing the density of functional groups on the surface, which allows for chains to attach. These two parameters allow for the PEG chain densities to be tuned from almost complete elimination of biofouling to densities that allow proteins, bacteria and cells to adhere.

## 2. Materials and Methods 

### 2.1. PEG Grafting

**Figure 1:**
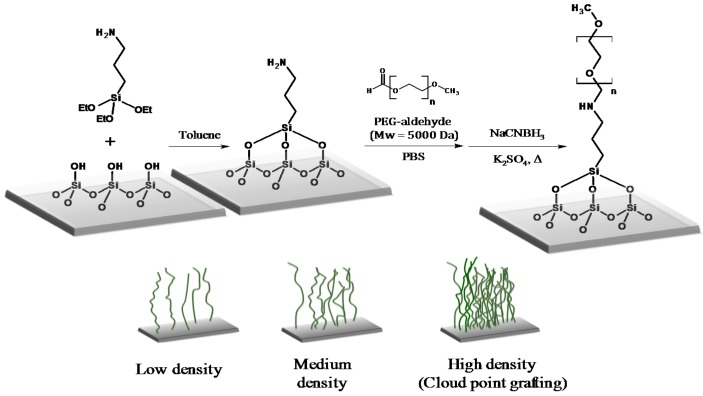
PEG-aldehyde grafting onto APTES modified silicon wafers.

Silicon wafers (1 cm^2^, M.M.R.C. Pty Ltd., Malvern, Australia) were cleaned with toluene in an ultrasonic bath for 5 min. Next, the Si wafers were rinsed with toluene, acetone and absolute ethanol (Sigma-Aldrich, Castle Hill, Australia) and dried under an N_2_ stream. After that, they were oxidised by UV/O_3_ chamber (BioForce Nanoscience, Salt Lake City, UT, USA) for 15 min. The thickness of the SiO_2_ layer was 13.8 Å measured by ellipsometry (J.A. Woollam Co., Inc., Lincoln, NE, USA). Then the silicon surface was reacted with three different concentrations of APTES (Sigma-Aldrich, Australia), i.e., 1%, 2% and 4% (*v*/*v*) in toluene for 30 min at room temperature, followed by rinsing with toluene, ethanol and Milli-Q water and were dried with N_2_ gas. The APTES modified Si wafers were placed in an oven at 110 °C for 15 min. After that, the APTES surfaces were reacted with PEG-aldehyde (*M*_w_ 5000, Sigma-Aldrich, Castle Hill, Australia) under different conditions to vary the PEG graft density. We used the “grafting to” approach because the graft density can be tuned by the solution conditions (polymer solubility), which was the aim of the study. PEG-aldehyde (2.5 mg/mL) was dissolved in 20 mL buffer solution (50 mL of 0.1 M sodium phosphate buffer at pH 7) containing either 0, 0.3 or 0.6 M (*w*/*v*) K_2_SO_4_. Then, 0.05 M NaCNBH_3_ (Sigma-Aldrich, Castle Hill, Australia) was added and the reaction kept in the oven for 4 h at 60 °C. They were then rinsed with Milli-Q water and dried with N_2_ gas [26] (Figure 1). 

### 2.2. Bovine Serum Albumin Adsorption 

The protein solution was prepared by dissolving bovine serum albumin (BSA) (cat#16000044, Invitrogen, Carlsbad, CA, USA) in phosphate buffer saline (PBS, pH 7.4) (Sigma-Aldrich, Castle Hill, Australia) at a concentration of 0.1%. Pieces of silicon wafer and APTES controls and PEG grafted substrates (1 cm × 1 cm) were immersed in Petri dishes (multi-12-well) with each well containing 1 mL of protein solution and were left in an incubator overnight at 37 °C. After that, samples were rinsed three times with MilliQ water to remove unadsorbed protein and dried with N_2_ [26].

### 2.3. Cell Attachment 

#### 2.3.1. Human Mesenchymal Stem Cells

Human mesenchymal stem cells were obtained from amniotic membranes (hAM-MSCs) during delivery by caesarean section. All samples were placed in 12-well plates for cell culture. Each surface was seeded with hAM-MSCs at a density of 1 × 10^4^ cells/cm^2^ in 2 mL media. hAM-MSC culture media was composed of mesenchymal stem cell growth medium (MSCGM™, Lonza, Mount Waverley, Australia) supplemented with MSCGM™ SingleQuots™ (Lonza, Australia). They were cultured for 24 h [27].

#### 2.3.2. Human MG63 Osteoblast-Like Cells

Human MG63 osteoblast-like cells (male) were purchased from ATCC (CRL-1427^TM^, Manassas, VA, USA) and passaged in a standard protocol according to supplier’s instruction. Cells with passage <30 from the supplier were used. Cells were seeded at a density of 1 × 10^4^ cells/cm^2^ in 12-well plates containing samples. Culture medium (2 mL), composed of 90% (*v*/*v*) Eagle’s Minimum Essential Medium (cat#30-2003, ATCC) and 10% (*v*/*v*) fetal bovine serum (cat#16000044, Invitrogen, Carlsbad, CA, USA), was added to each well, and then the cells were cultured for 24 h [28].

### 2.4. Pseudomonas Aeruginosa Culture

Bacterial attachment experiments were performed on all surfaces. The PEG grafted surfaces including controls (Si wafer and APTES surfaces) were transferred into 12-well plates. *P. aeruginosa* (ATCC 15692) was streaking on an agar plate with 1.5% nutrient agar for 18 h at 37 °C. A single bacterial colony was transferred from the agar plate into 30 mL of Müller–Hinton Broth in a 50 mL tube. The culture was incubated for 18 h at 37 °C by shaking at 250 rpm in a shaking incubator (RATEK, Boronia, Australia). Next day, the culture media was removed from the incubator and transferred into 1 mL sterile Eppendorf tubes. The pellet was obtained from the culture media by spinning down at 8000 rpm for 3 min, using a centrifuge (DYNAMICA, Newport Pagnell, UK). The upper solution was removed with a pipette and disposed in the bleach water mixture waste. The pellets were resuspended in 1 mL MHB, this step was repeated for a total of three washes. Once the cells were resuspended for the final time, 2 mL of sterile MHB and 20 µL of the resuspended cells (1 in 100 dilutions) were pipetted into a sterile 14 mL tube. The culture was then incubated for 2 h at 37 °C in a shaking incubator at 250 rpm. MHB medium was pre-warmed to 37 °C in a heat bath whilst the culture was growing. The cells were diluted in warm media and mixed by pipetting up and down (30 µL of cells and 2970 µL of warm media) in 12-well plates, the culture optical density, OD600 nm = 0.1. Finally, 2 mL of the final solution was transferred onto the Si wafer, APTES and PEG grafted surfaces within the 12-well plates and incubated at 37 °C for 2 h. Then, the bacterial solution was removed from surfaces carefully, and the substrates were washed three times with PBS [29].

### 2.5. Surface Characterisation

#### 2.5.1. X-Ray Photoelectron Spectroscopy (XPS)

XPS spectra were recorded using an AXIS Nova spectrometer (Kratos Ltd, Telford, UK) equipped with monochromatic aluminium source (Al kα 1486.6 eV) operating at 150 W. The total pressure in the sample analysis chamber during analysis was of the order of 10–9 mbar. Three different data points on each sample were assessed. An elliptical area with approximate dimensions of 0.7 mm × 0.4 mm was analysed on each sample. All elements were identified from survey spectra (acquired at pass energy of 160 eV). To observe more comprehensive information such as oxidation states, chemical structure high-resolution spectroscopy was performed from individual peaks at a detector pass energy of 20 eV. CasaXPS Version 2.3.15 software (Casa Software Ltd, Teignmouth, UK) was used to analyse data generated using sensitivity factors supplied with the instrument. High resolution C 1s spectra were fitted with Gaussian-broadened Lorentzian functions after linear background subtraction the spectra were referenced to the C–C/C–H binding energy at 285.0 eV.

The overlayer thicknesses were calculated based on attenuation length of the Si wafer signal using the Equation (1) [30,31]

(1) $$-Z=\lambda \cos\theta \;\ln\left(\frac{I}{I_{0}}\right)$$

where *Z* is the overlayer thickness, λ is the inelastic mean free path of photoelectrons (3.85 nm), θ is the take-off angle (0°), *I*_0_ and *I* are the bare substrate and thin film coating intensities.

#### 2.5.2. Ellipsometry

The overlayer thickness of APTES and PEG layers were measured using a M-2000XI spectroscopic ellipsometer (J.A. Woollam Co., Inc., Lincoln, NE, USA) equipped with Deuterium and Quartz Tungsten Halogen lamps (210.9–1687.7 nm) with incident angles of 50° and 70°. The thickness was determined using the Cauchy model by fitting the experimental data to the model using CompleteEASE software (J.A. WOOLLAM CO., INC., Lincoln, NE, USA).

#### 2.5.3. Contact Angle Measurements

The surface wettability of the substrates was measured using a contact angle goniometer (FTA1000 instruments, Schaumburg, IL, USA) by determining the static sessile drop of Milli-Q water (0.5 μL). Three spots were analysed for each sample (*n* = 3).

#### 2.5.4. Atomic Force Microscopy (AFM)

Surface roughness was measured using a Multimode VIII (Bruker, Billerica, MA, USA) atomic force microscope (AFM), and a Nanoscope V controller (Bruker, USA) in tapping mode in air. Scan sizes of either 1 or 5 µm^2^ were recorded using antimony (n) doped silicon cantilevers with a spring constant of 40 N/m (RTESPA-300, Bruker) and resonant frequency of approximately 300 kHz. The resolution of AFM images was recorded with 512 × 512 pixels. Post imaging of all scans was flattened (1st Order) and the route mean squared (*R*_q_) and arithmetical (*R*_a_) roughness values calculated in the Nanoscope 8.15 analysis software (Bruker). Roughness values are quoted as a mean of triplicate scans and the error is quoted as plus or minus one standard deviation. 

### 2.6. Cell Analysis

#### Epifluorescence Imaging

Cell morphologies was studied using epifluorescence microscopy (Eclipse Ti-E microscope, Nikon, Tokyo, Japan). Samples were fixed with 4% (*v*/*v*) paraformaldehyde/PBS for 30 min., permeabilised by 0.2% Triton X-100/PBS (PBST) for 15 min, then washed with PBS. Cell nuclei and F-actin were stained using DAPI (100 nM in PBS) and Phalloidin-TRITC (500 nM in PBS) for 1 h, respectively. Fluorescent stains were imaged at excitation/emission wavelengths of 346/442 nm for DAPI and 555/565 nm for Phalloidin-TRITC. National Institutes of Health ImageJ 1.48v software (NIH, Bethesda, Bethesda, Rockville, MD, USA) was used to calculate the number of attached cells [28].

### 2.7. Bacterial Analysis

#### 2.7.1. Epifluorescence Imaging

To obtain fluorescent images of attached bacteria the rinsed samples were first fixed using 2 mL of 3.7% (*v*/*v*) paraformaldehyde/PBS, followed by three times rinsing with PBS buffer, and finally, the bacteria were stained with 100 nM 4,6-diamidino-2 phenylindole (DAPI, cat#D9542; Sigma-Aldrich, Australia) for 30 min [32]. Subsequently, the DAPI solution was removed, and samples were gently rinsed two times with 2 mL of PBS for 5 min each. The samples were kept in PBS buffer for imaging. Fluorescent images were captured using an inverted epifluorescence microscopy with 20× lens (Eclipse Ti-Nikon, Instruments, Inc., Tokyo, Japan). The area covered by DAPI-stained bacteria and each image was analysed using National Institutes of Health ImageJ 1.48v software. Ten images (20×: 449.35 µm^2^) from two surfaces of each sample were analysed (*n* = 6) [29].

#### 2.7.2. Scanning Electron Microscopy (SEM)

SEM was used to image the attached bacteria that were first fixed using 3.7% (*v*/*v*) paraformaldehyde as described in 2.7.1. These samples were rinsed with Milli-Q water and dried at room temperature. Prior to imaging, all samples were sputter coated with approximately 15 nm Au. Then, all samples were analysed using ZEISS SUPRA 40 SEM (VP Carl Zeiss SMT, Oberkochen, Germany) at 3–5 keV.

## 3. Results

### 3.1. XPS Analysis and Ellipsometry of PEG Layers

The XPS survey spectra were used to identify and quantify the elements present on surfaces; the atomic compositions are shown in Table 1. In general, the atomic percentage of carbon increased and silicon peak intensity decreased with increasing PEG grafting density. At the same time, the terminal nitrogen of APTES (%At.) decreased gradually with increasing coating thickness.

**Figure 2:**
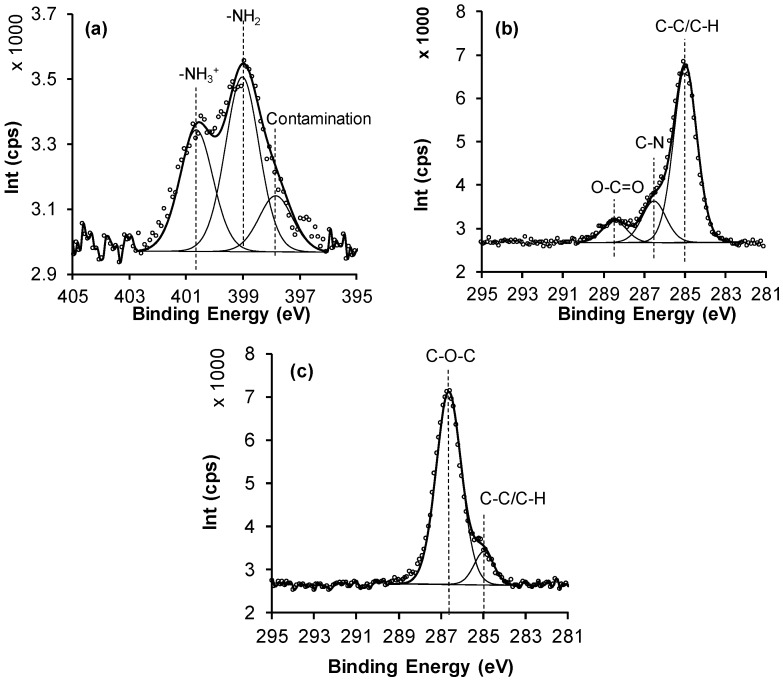
Selected XPS spectra for modified surfaces: (**a**) N 1s spectrum of 4% APTES, (**b**) C 1s spectrum of 4% APTES, and (**c**) C 1s spectrum of PEG grafted under ”cloud point” conditions.

In order to determine chemical state of the individual elements, high resolution XPS analysis was performed. Of particular interest is the N 1s spectra for the wafers after APTES grafting (Figure 2a), which predominantly shows nitrogen in the form of primary amine groups with the peak at 399 eV; however, there is also a weaker peak at 400.5 eV assigned to a protonated amine and a lower binding energy peak that we assign to contamination [33]. Furthermore, for the XPS, C 1s of a typical APTES surface (Figure 2b) showed peaks at 285.0 eV and 285.8 eV, which are assigned to C–C/C–H and C–N components, respectively [34]. 

After PEG grafting, the C 1s spectra showed peaks at 285.0 eV and in particular 286.5 eV confirming the presence of ether C–O and C–C components, respectively (Figure 2c). The etheric component subsequently increases as the K_2_SO_4_ content in the solution increased, with a subsequent decrease in nitrogen signal as more PEG chains were grafted to the APTES surface (Table 1) [35,36]. Grafting PEG at the lower critical solution temperature (LCST) has been used by researchers to give low protein adsorption [37,38,39]. The covalent attachment of PEG to the APTES surface was performed under conditions of different concentration of K_2_SO_4_ and it is evident that grafting at a high content of K_2_SO_4_ at 60 °C results in more attached PEG that is confirmed by XPS signal related to an increase in PEG density of tethered chains. The concentration of salt affected directly the grafted density of the polymer (Table 1), where increasing the concentration of salt from 0 to 0.6 M at 60 °C results in a lowering of solubility of PEG and increases the grafted PEG density.

**Figure 3:**
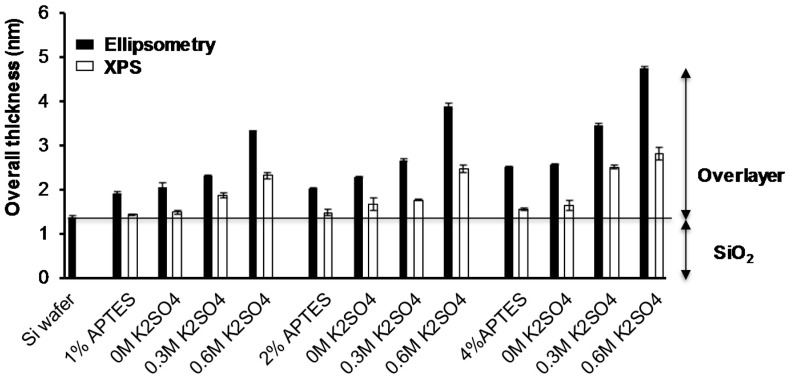
Overlayer thickness of Si wafer, APTES and PEG grafted surfaces measured using ellipsometry and XPS. Data shown: mean ± STD of the mean (*n* = 3).

In order to obtain information about the relative density of PEG chains grafted to the APTES surfaces, the total overlayer thicknesses were determined using both XPS and ellipsometry measurements and the results are shown in Figure 3. The thicknesses of all modified surfaces increased with increase the PEG layers at different conditions, higher APTES and salt concentration raised the overlayer thickness of all modified surfaces.

### 3.2. Water Contact Angle Measurements and Atomic Force Microscopy

The wettability of the modified surfaces is shown in Table 2. It is clear that, after APTES grafting, the surfaces becomes more hydrophobic compared to the control Si wafer. Interestingly, the 4% APTES surface is more hydrophobic than the 1% and 2% APTES. This could be attributed to the higher N content and a thicker silane coating. However, after PEG grafting, there is a decrease in sessile drop contact angle that is minimised at the highest graft of density of PEG on all APTES surfaces. The value of 30 degrees is in accordance with that previously published for PEG surfaces [40].

**Figure 4:**
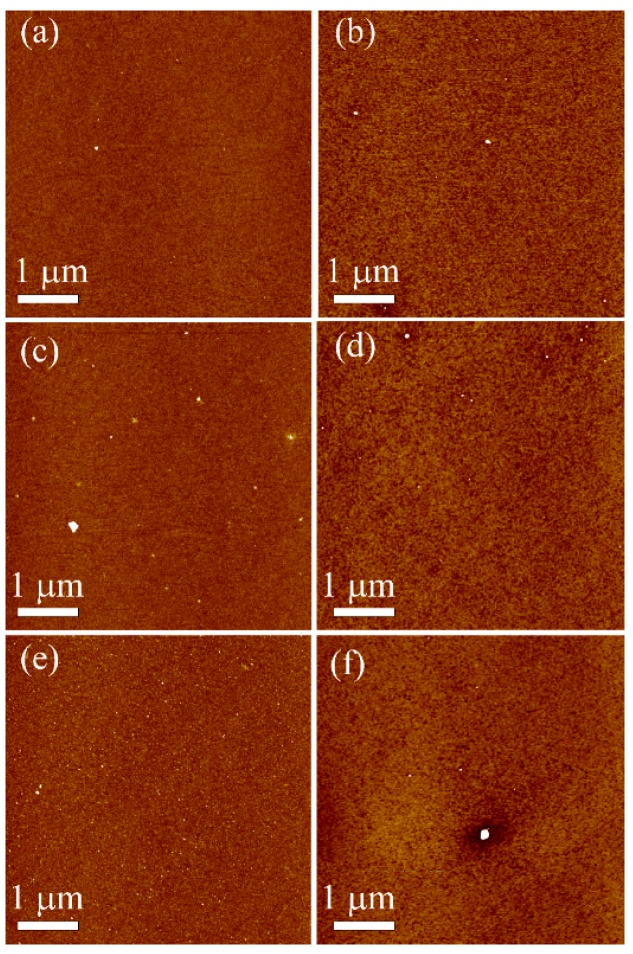
AFM topography images recorded for: (**a**) 1% APTES, (**b**) 1% APTES_PEG/0.6 M K_2_SO_4_/60 °C, (**c**) 2% APTES, (**d**) 2% APTES_PEG/0.6 M K_2_SO_4_/60 °C, (**e**) 4% APTES, and (**f**) 4% APTES_PEG/0.6 M K_2_SO_4_/60 °C (OD600 nm = 0.48). All *z*-scales were equal to 5 nm. (Higher resolution images can be seen in Figure S1).

AFM measurements were acquired to provide information about the topography and roughness of modified surfaces. Comparing the topography images of the APTES control and grafted PEG at the cloud point (0.6 M K_2_SO_4_ at 60 °C), it can be seen that the surfaces vary in the cluster sizes that are known to form with silane modification [41] (Figure 4), and these appeared to increase after PEG grafting. Additionally, the overall topography for the grafted PEG substrates is subtly different, with the PEG functionalized films having a more porous and uneven surface topography (this can be more clearly seen in the higher resolution scans in the supporting information). Quantification of both the geometric average roughness (*R*_q_) and arithmetical roughness (*R*_a_) revealed a small increase upon PEG grafting for all three concentrations of APTES (Table 2). *R*_q_ was seen to increase to a greater extent than *R_a_* (e.g., for 4% APTES_PEG, the mean *R*_q_ increases by 0.45 nm but the *R_a_* only increases by 0.05 nm), and this is due to the fact that the value of *R*_q_ is much more sensitive to the large clusters that appear on the surface after PEG grafting (see Figure 4f). The small increases in roughness and changes in topography upon PEG grafting are likely due to increased disorder on the surface due to the significantly longer polymer PEG chains compared to the short chain well-ordered APTES self-assembled monolayers. However, the increase in average surface roughness after PEG grafting on the APTES surfaces was relatively small (Table 2), suggesting the formation of uniform SAMs and PEG layers [34].

### 3.3. Protein Adsorption onto the Grafted PEG Layers

**Figure 5:**
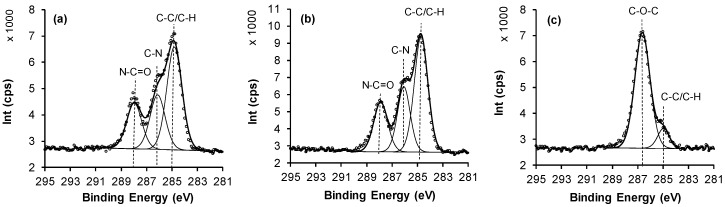
C 1s XPS spectra after BSA adsorption to the: (**a**) Si wafer (control), (**b**) the APTES surfaces and (**c**) high density PEG layer surface.

The ability of PEG layers with specific densities to adsorb BSA was determined by XPS, ellipsometry and water contact angle measurements as shown in Table 3 and Table 4. Table 3 shows the XPS elemental composition for all the surfaces after exposed to BSA and demonstrates that adsorption is confirmed by the increases in %N and amide component (%N–C(1s)=O) obtained from curve-fitted C 1s spectra, which arise from the amino acid structure of the protein. Selected C 1s spectra for the Si wafer, 4% APTES and high graft density PEG (0.6 M K_2_SO_4_/60 °C) surfaces are shown in Figure 5. For the Si wafer and APTES surfaces, the %N increases indicate that the level of protein adsorption is close to a monolayer, which is expected for surfaces unable to repel proteins. For BSA adsorption to the PEG surfaces with variable density of chains, it is clear that there is a trend in the ability of the PEG surfaces to repel BSA. The results show that both the %N and amide content decreases from low to medium to high PEG density, and when grafting is performed at the cloud point (0.6 M K_2_SO_4_/60 °C), it appears that BSA is fully repelled from the surface since little change to the atomic composition occurs after adsorption. According to literature, a high PEG minimises protein adsorption [26,42], and this was confirmed during this study, while a low PEG density is able to trap some of the BSA molecules on the surface.

Water contact angles increased after protein adsorbed on controls and modified surfaces and it decreased when the PEG density increase, confirming the hydrophilicity of PEG modified surfaces (Table 4).

### 3.4. Human Mesenchymal Stem Cell, MG63 Osteoblast-Like Cell and P. aeruginosa Attachment

**Figure 6:**
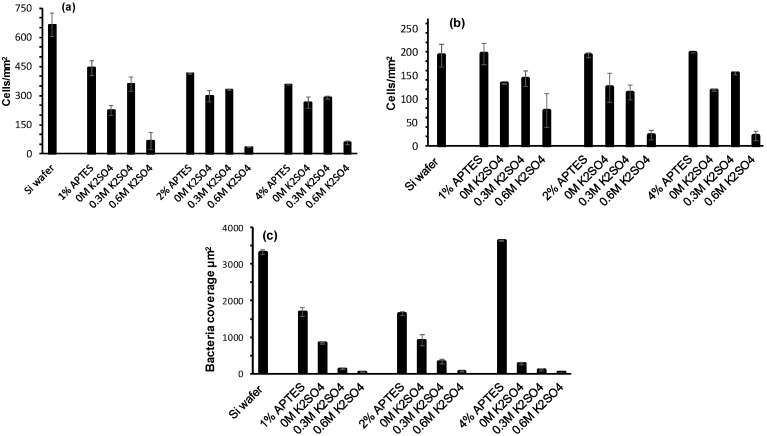
(**a**) Human mesenchymal stem cell, (**b**) MG63 osteoblast-like cell, and (**c**) *P. aeruginosa* attachment to Si wafers, APTES and PEG surface grafted under different conditions.

**Figure 7:**
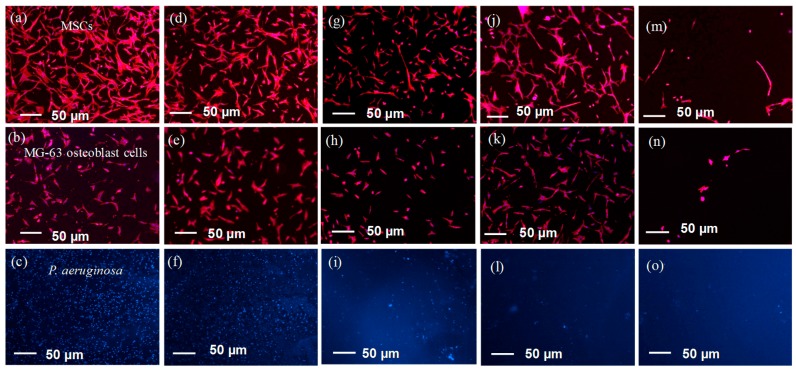
Epifluorescence images of human mesenchymal stem cell, MG63 osteoblast-like cell and *P. aeruginosa* attachment to PEG grafted under different conditions: (**a**–**c**) Si wafer control, (**d**–**f**) 4% APTES, (**g**–**i**) 4% APTES_PEG 0 M K_2_SO_4_, (**j**–**l**) 4% APTES_PEG 0.3 M K_2_SO_4_, and (**m**–**o**) 4% APTES_PEG 0.6 M K_2_SO_4_.

To determine the influence of PEG grafted surfaces on both the ability to control mammalian cell attachment as well as repelling bacteria, assays were performed with human mesenchymal stem cells (MSCs), MG63 osteoblast-like cells and *P. aeruginosa*. For mammalian cell attachment and proliferation, all modified surfaces were exposed to cells for 24 h followed by staining using Phalloidin-TRITC and DAPI [28]. Figure 6a,b show the level of cell attachment to each surface and Figure 7a,b show the fluorescent images from which the quantitative data was obtained. Firstly, the results demonstrate that there is a difference in attachment of either MSCs or MG63 osteoblasts to each type of surface. For MSCs, the control Si wafer has the highest number of cells attached followed by the APTES surfaces. On the other hand, the level MG63 osteoblast cell attachment is the same for the Si wafer and APTES surfaces. 

For the PEG grafted surfaces, the degree of MSC and MG63 osteoblast attachment is somewhat variable. For the cloud point grafted PEG, the numbers of MSCs and MG63 osteoblast cell attaching is minimised in line with a high-density polymer brush layer’s ability to repel attachment. However, for the low- and medium-density PEG layers, there are interesting observations. In this case, there are slightly more MSCs adhering to the medium density PEG layer surface compared to the low density PEG layer surface, whereas, for MG63 osteoblasts, this only occurs for the medium density PEG surface grafted to 4% APTES. We attribute the higher cell density to serum protein adsorption, where the possibility exists for the proteins to become trapped and remain more active (less denatured) in the hydrophilic PEG layers of medium density. The feature that is variable between the cell types is the morphology on the PEG layers (Figure 7a,b). The MSCs appear highly elongated on the high density PEG surface compared to the much more rounded cell morphology for the MG63 osteoblasts. For all of the other surfaces, both cell types have a range of morphologies from well spread to elongated, most likely due to accessibility to adsorbed proteins on those surfaces or within the PEG chains. 

In order to study the influence of the PEG layers on bacterial attachment, all surfaces were exposed to *P. aeruginosa* at 37 °C for 2 h, followed by DAPI staining and epifluorescence imaging to determine the levels of attached bacteria. The data in Figure 6c show the surface coverage of bacteria determined using ImageJ analysis of the fluorescent images. The control Si wafer and the 4% APTES surfaces showed the highest numbers of attached bacteria, followed by the 1 and 2% APTES. The trend for APTES can be attributed to the level of amine groups on the surface with 4% APTES having a higher N content as determined by XPS. It is likely that the 4% APTES surface has more positively charged amine groups that can better attract the oppositely charged *P. aeruginosa*, as we previously demonstrated [29]. Interestingly, there were high numbers of bacteria attached to the Si wafer surface, which starts off being negatively charged on account of the SiO_2_ oxide layer. One would expect electrostatic repulsion of *P. aeruginosa*, but this demonstrates that others factors are responsible for facilitating bacterial attachment other than electrostatics, with the possibility that growth media components adsorbing and providing a conditioning film for the attachment. 

**Figure 8:**
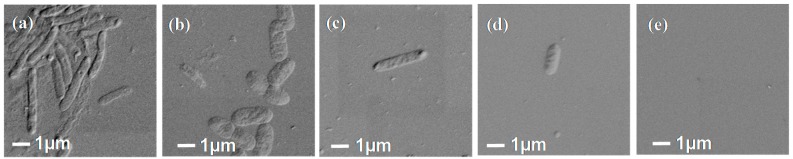
SEM images of *P. aeruginosa* attachment to: (**a**) Si wafer control, (**b**) 4% APTES, (**c**) 4% APTES_PEG 0 M K_2_SO_4_, (**d**) 4% APTES_PEG 0.3 M K_2_SO_4_, and (**e**) 4% APTES_PEG 0.6 M K_2_SO_4_.

*P. aeruginosa* attachment to the grafted PEG layers made under different solubility conditions showed quite a different trend to the MSC and MG65 osteoblast attachment in that there was a clear trend in attachment density with increasing PEG coverage on the surface with negligible bacterial attachment occurring on the cloud point grafted PEG surface (Figure 6c). The fluorescent images in Figure 7c–o and the SEM images in Figure 8 also confirm the trend in attachment to the PEG surfaces with few bacteria attached at high PEG density in agreement with a previous study [14].

## 4. Discussion

In this study, we have changed the PEG chain grafting density in several ways by using different APTES concentrations to generate reactive surfaces with different concentrations of amine groups and by grafting PEG under different solution conditions to vary the surface density of polymer chains. These surfaces were then tested in bioassays that included studying protein adsorption, mammalian cell (MSC and MG63 osteoblast) and bacterial (*Pseudomonas aeruginosa*) attachment. 

The highest PEG coverage was achieved by grafting at the ‘cloud point’ (CP) of the PEG. This is attributed to a reversible phase change of PEG molecules under these specific conditions due to rupturing the hydration surrounding the PEG chains through anion (SO_4_^2−^) interactions with the PEG molecules that is enhanced at high temperatures. Under these poor solvent conditions, there is a reduction in the repulsion between the PEG chains and a lowering of the radius of gyration of the individual molecules, which results in a maximum packing density, generating very high PEG graft densities if there is a sufficiently high concentration of reactive functional groups on the modified surface [26]. In addition, different densities of PEG were obtained depending on different concentrations of APTES used for surface functionalisation, i.e., a variable concentration of surface NH_2_ groups was generated, and, at the highest density of APTES, a sufficiently high PEG density was achieved when combined with CP grafting (0.6 M K_2_SO_4_ and 60 °C). Previously, low protein adsorption was observed by grafting of PEG under lower critical solution conditions [37,38,39,43]. On the other hand, many studies of PEG grafting have used conditions in which the PEG chains were highly solvated and therefore not able to produce a high density of grafting; those studies observed a reduction, but not exclusion, of protein adsorption on the surface, but complete elimination of adhesion by PEG is theoretically possible at high chain densities [44,45,46].

According to XPS and ellipsometry measurements, the overlayer thickness of grafted PEG increased gradually depending on salt concentration, although the absolute values obtained from XPS and ellipsometry were not the same, the trends in thickness variations under different condition were the same. The discrepancies between the two techniques are most likely due to the different assumptions used by both methods. .

With respect to the protein adsorption the results showed that, at the highest polymer chain density, minimal BSA was achieved. Since all of the forces emanating from the underlying substrate that are responsible for protein adsorption, i.e., hydrogen bonding, van der Waals, and electrostatic, are screened by the neutral, non-ionic steric stabilising PEG brushes, this result is not unexpected. As the PEG graft density is reduced, the BSA molecules are capable of penetrating the interstitial spaces between the PEG chains since the distance between adjacent chains is smaller than the dimensions of the BSA molecules. In order to compensate for this, a large radius of gyration of the random coil PEG molecules or higher molecular weight would be needed for a given graft density. On the control Si wafer and APTES surfaces, functional groups are present such as the Si wafer oxygen groups that allow the interaction between the protein and the surface by hydrogen bonding and ionic interactions [47], and the NH_2_ and hydrophobic groups on the APTES layers that allow ionic and hydrophobic interactions to facilitate BSA adsorption. The PEG molecules shield these charged groups decreasing protein adsorption on the modified surfaces [19], and the combination of steric hindrance is the reason for decreases of protein adsorption on the PEG layers [44,48,49]. 

With respect to human mesenchymal stem and MG63 osteoblast-like cells, attachment was observed on both PEG modified and unmodified surfaces and the levels of attachment were reduced by PEG and minimised at maximum PEG chain density, which was previously confirmed through other studies [50,51]. The decrease in protein adsorption correlates to the cell adhesion for the high density PEG layers, but, at low and medium densities, there is less of a trend in cell repulsion and a difference between cell types. The serum proteins in the cell culture medium contain a range of proteins that can potentially adsorb to the low and medium density PEG surfaces facilitating the attachment levels observed with the reactivity most likely being cell-type dependent, hence the differences in cell density and cell morphology observed. Moreover, studies have shown that cell adhesion is enhanced on hydrophilic surfaces [52,53], while proteins adsorb to a greater extent on hydrophobic surfaces [53,54], and it is clear that interfacial proteins are responsible for cell behaviour [55,56,57]. Therefore, we attribute the cell attachment on the PEG grafted layers to serum protein adsorption. Furthermore, the steric factors exhibited by the PEG layers, with a lack of ionic interactions between the cells and PEG chains, plays an important role in reducing the cell attachment. The PEG molecules exist in different configurations depending on their surface density with chains occupying a specific volume because they exist as random coils when attached to a surface by a single bond [50,58]. These different PEG densities therefore have the potential to adsorb proteins of different size, with different concentrations, and help keep them in a state that maximises the interactions with cell surface integrins, potentially providing a platform to optimise cell function such as controlled proliferation and differentiation. 

For the bacterial attachment studies, the Si wafer and APTES surfaces (controls) showed the highest coverage of bacteria, and the adhesion in part can be attributed again to hydrogen bonding and ionic interactions. For the APTES layers, adhesion is enhanced by electrostatic attraction between positively charged amine groups and the negatively charged extracellular polysaccharides (EPS) of *Pseudomonas aeruginosa* [59,60,61]. On the other hand, the PEG modified surfaces showed less bacteria compared to Si and APTES surfaces and this is attributed to the hydrophilicity of covalently grafted PEG, which is important to overcome the possible deleterious adverse responses that bacteria cause when they colonize surfaces. These results are similar to previously reported work for short-term reduction of bacterial adhesion through PEG layers [14,62,63]. Interestingly, the bacteria attachment trend was different to that of the mammalian cells, which indicates that the latter are more sensitive to subtle variations in PEG density and subsequent protein adsorption. It would be interesting in future work to observe if bacterial attachment was enhanced after the PEG surfaces were exposed to serum proteins and if mammalian cell culture under serum free conditions resulted in a trend similar to the bacterial attachment results. Clearly, our understanding of how PEG and other non-fouling surfaces function and the interplay between protein adsorption in cell and bacterial attachment on those surfaces requires more research.

## 5. Conclusions

In this study, we modified silicon wafer surfaces with an amino functionalised silane (APTES) that was used to covalently attach PEG chains of different densities aimed at enhancing protein adsorption in order to promote human cell attachment and at the same time resist bacterial adhesion with the potential to eliminate infection on implants. We demonstrated in a systematic way that the graft density of PEG depended on the salt concentration (K_2_SO_4_) of the solution, and as the salt content increased, the polymer density increased and reached the highest density at 0.6 M/60 °C. It is noted that low protein adsorption was observed on a high polymer density surface and both human mesenchymal stem and MG63 osteoblast-like cells attachment was minimised, but at low and medium PEG densities, attachment was increased but in a cell and surface dependent manner. In bacterial adhesion studies, the trend of reduction in *P. aeruginosa* attachment followed that of the PEG chain density demonstrating the universal nature of the PEG grafted layers. The results open up the possibility of having biomaterial surfaces that can facilitate attachment and growth of mammalian but minimise the risk of infection by repelling bacterial attachment.

## Figures and Tables

**Table 1:** XPS elemental compositions recorded on PEG grafted surfaces under different conditions at 60 °C.

Sampler	%C *	%O *	%N *	%Si *	%C ether/COH
Si wafer (control)	8.6 ± 0.6	35.1 ± 0.6	0	56.3 ± 0.0	0
1% APTES (control)	13.4 ± 0.2	33.0 ± 0.0	0.6 ± 0.0	52.9 ± 0.3	2.9 ± 0.3
1% APTES + PEG/0 M K_2_SO_4_	14.1 ± 0.0	33.3 ± 0.8	0.5 ± 0.0	52.1±0.8	7.9 ± 0.1
1% APTES + PEG/0.3 M K_2_SO_4_	21.0 ± 0.5	32.1 ± 0.2	0.3 ± 0.4	46.6 ± 0.8	14.7 ± 0.6
1% APTES + PEG/0.6 M K_2_SO_4_	34.0 ± 0.1	32.1 ± 0.2	~0	34.0 ± 0.2	27.7 ± 1.2
2% APTES (control)	13.7 ± 0.4	31.8 ± 0.5	1.2 ± 0.3	53.3 ± 0.5	2.9 ± 0.1
2% APTES + PEG/0 M K_2_SO_4_	16.2 ± 1.1	33.2 ± 0.3	0.9 ± 0.1	49.7 ± 0.8	10.0 ± 0.6
2% APTES + PEG/0.3 M K_2_SO_4_	25.3 ± 1.5	32.1 ± 0.0	0.7 ± 0.1	41.9 ± 1.4	19.8 ± 1.2
2% APTES + PEG/0.6 M K_2_SO_4_	36.8 ± 0.7	32.4 ± 0.2	~0	30.9 ± 0.5	30.3 ± 1.1
4% APTES (control)	18.9 ± 0.2	31.3 ± 0.3	2.2 ± 0.1	47.8 ± 0.2	1.8 ± 0.2
4% APTES + PEG/0 M K_2_SO_4_	23.3 ± 0.8	32.0 ± 0.3	1.5 ± 0.1	43.3 ± 0.4	15.3 ± 0.8
4% APTES + PEG/0.3 M K_2_SO_4_	32.0 ± 0.2	31.9 ± 0.3	1.3 ± 0.0	34.9 ± 0.5	21.6 ± 0.6
4% APTES + PEG/0.6 M K_2_SO_4_	43.1 ± 0.7	31.8 ± 0.0	1.0 ± 0.1	24.1 ± 0.8	35.9 ± 2.2

* Taken from C 1s, O 1s, N 1s, Si 2p spectra.

**Table 2:** Water contact angles and surface roughness values for APTES and PEG grafted silicon wafer surface under various ionic strength conditions at 60 °C.

Sample	Water Contact Angle (θ)	Roughness (*R*_q_) (nm)	Roughness (*R*_a_) (nm)
Si wafer (control)	26 ± 0.4	0.18 ± 0.02	0.13 ± 0.01
1% APTES (control)	38.5 ± 2.2	0.19 ± 0.03	0.16 ± 0.05
1% APTES + PEG/0 M K_2_SO_4_	36.9 ± 1.5		
1% APTES + PEG/0.3 M K_2_SO_4_	34.7 ± 1.6		
1% APTES + PEG/0.6 M K_2_SO_4_	31.3 ± 1.3	0.30 ± 0.06	0.19 ± 0.01
2% APTES (control)	43.4 ± 0.6	0.26 ± 0.06	0.14 ± 0.01
2% APTES + PEG/0 M K_2_SO_4_	38.1 ± 1.9		
2% APTES + PEG/0.3 M K_2_SO_4_	31.4 ± 0.7		
2% APTES + PEG/0.6 M K_2_SO_4_	29.4 ± 0.9	0.35 ± 0.22	0.18 ± 0.01
4% APTES (control)	57 ± 3.1	0.26 ± 0.06	0.16 ± 0.01
4% APTES + PEG/0 M K_2_SO_4_	41.3 ± 2.5		
4% APTES + PEG/0.3 M K_2_SO_4_	31.0 ± 0.6		
4% APTES + PEG/0.6 M K_2_SO_4_	29.5 ± 0.7	0.69 ± 0.75	0.21 ± 0.03

**Table 3:** XPS elemental compositions for BSA adsorbed to Si wafer, APTES and PEG surfaces grafted under different conditions at 60 °C.

Sampler	%C *	%O *	%N *	%Si *	%N 1s Amide
Si wafer (control)	39.8 ± 1.1	22.5 ± 0.05	8.8 ± 0.3	28.9 ± 1.4	0
1% APTES (control)	32.2 ± 0.5	24.5 ± 0.3	6.8 ± 0.3	36.3 ± 0.5	7.1 ± 0.1
1% APTES + PEG/0 M K_2_SO_4_	31.8 ± 0	24.7 ± 0.1	6.9 ± 0.4	36.5 ± 0.2	7.0 ± 0.1
1% APTES + PEG/0.3 M K_2_SO_4_	31.9 ± 0.4	25.7 ± 0.3	5.8 ± 0	36.5 ±0.7	5.9 ± 0.1
1% APTES + PEG/0.6 M K_2_SO_4_	22.2 ± 1.1	30.3 ± 0.2	1.0 ± 0.1	46.5 ± 1	1.2 ± 0.1
2% APTES (control)	35.2 ± 0	24.7 ± 0.2	7.3 ± 0.1	32.7 ± 0.1	7.6 ± 0.1
2% APTES + PEG/0 M K_2_SO_4_	33.8 ± 1.9	24.6± 0.5	7 ± 0.5	34.5 ± 1.9	7.2 ± 0.3
2% APTES + PEG/0.3 M K_2_SO_4_	24.2 ± 0.6	26.6 ± 0.2	4.1 ± 0.1	45.1 ± 0.3	4.1 ± 0.4
2% APTES + PEG/0.6 M K_2_SO_4_	19.4 ± 0.3	30.1 ± 0.2	0.7 ± 0.1	49.9 ± 0.2	1.1 ± 0.1
4% APTES (control)	46.1 ± 0.5	23.1 ± 0.1	10.4 ±0.1	20.5 ± 0.5	9.7 ± 0.1
4% APTES + PEG/0 M K_2_SO_4_	39.1 ± 0.4	22.8 ± 0.7	8.0 ± 0.1	30.1 ± 0.2	7.9 ± 0.1
4% APTES + PEG/0.3 M K_2_SO_4_	23.6 ± 1.7	27.8 ± 0.3	3.1 ± 0.2	45.5 ± 1.5	3.3 ± 0.05
4% APTES + PEG/0.6 M K_2_SO_4_	23.0 ± 0.1	30.7 ± 0.1	1.2 ± 0.3	45.2 ± 0.1	0.7 ± 0.02

* Taken from C 1s, O 1s, N 1s, Si 2p spectra.

**Table 4:** Water contact angle and ellipsometry measurements of BSA adsorbed on modified surfaces at 60 °C.

Sample	Water Contact Angle (θ)	Ellipsometry (nm)
Si wafer (control)	52.1 ± 4.5	3.6 ± 0.4
1% APTES (control)	55.2 ± 2.3	3.6 ± 0.4
1% APTES + PEG/0 M K_2_SO_4_	45.3 ± 2.2	4.1 ± 0.3
1% APTES + PEG/0.3 M K_2_SO_4_	42.5 ± 1.6	3.8 ± 0.1
1% APTES + PEG/0.6 M K_2_SO_4_	36.4 ± 3.3	3.5 ± 0.04
2% APTES (control)	50.2 ± 1.5	3.0 ± 0.7
2% APTES + PEG/0 M K_2_SO_4_	45.0 ± 2.3	3.8 ± 0.2
2% APTES + PEG/0.3 M K_2_SO_4_	35.0 ± 1.1	3.4 ± 0.1
2% APTES + PEG/0.6 M K_2_SO_4_	30.3 ± 3.3	3.9 ± 0.1
4% APTES (control)	72.1 ± 1.6	4.1 ± 1.0
4% APTES + PEG/0 M K_2_SO_4_	60.3 ± 1.8	3.9 ± 0.5
4% APTES + PEG/0.3 M K_2_SO_4_	41.2 ± 2.4	4.0 ± 0.4
4% APTES + PEG/0.6 M K_2_SO_4_	32.1 ± 1.3	5.1 ± 0.1

## Supplementary Materials

The following are available online at www.mdpi.com/2073-4360/9/8/343/s1.
