# Supplementary material for: Tuning the Density of Poly(ethylene glycol) Chains to Control Mammalian Cell and Bacterial Attachment
Source: Polymers (Basel). 2017 Aug 5;9(8):343. doi: 10.3390/polym9080343 (PMC6418490; doi:10.3390/polym9080343)
Supplement: Supplementary file 1 [file polymers-09-00343-s001.docx]

**SUPPORTING INFORMATION**

Tuning the density of poly(ethylene glycol) chains to control mammalian cell and bacterial attachment

Ahmed Al-Ani^1^, Hitesh Pingle^1^, Nicholas P Reynolds^2^, Peng-Yuan Wang^1^*, Peter Kingshott^1^*

^1^ Department of Chemistry and Biotechnology, School of Science, Faculty of Science, Engineering and Technology, Swinburne University, Hawthorn, Vic. 3122, Australia

^2^ ARC Training Centre for Biodevices, Faculty of Science, Engineering and Technology, Swinburne University of Technology, Hawthorn, Vic 3122, Australia

***** Correspondence: pengyuanwang@swin.edu.au, ‎pkingshott@swin.edu.au

**
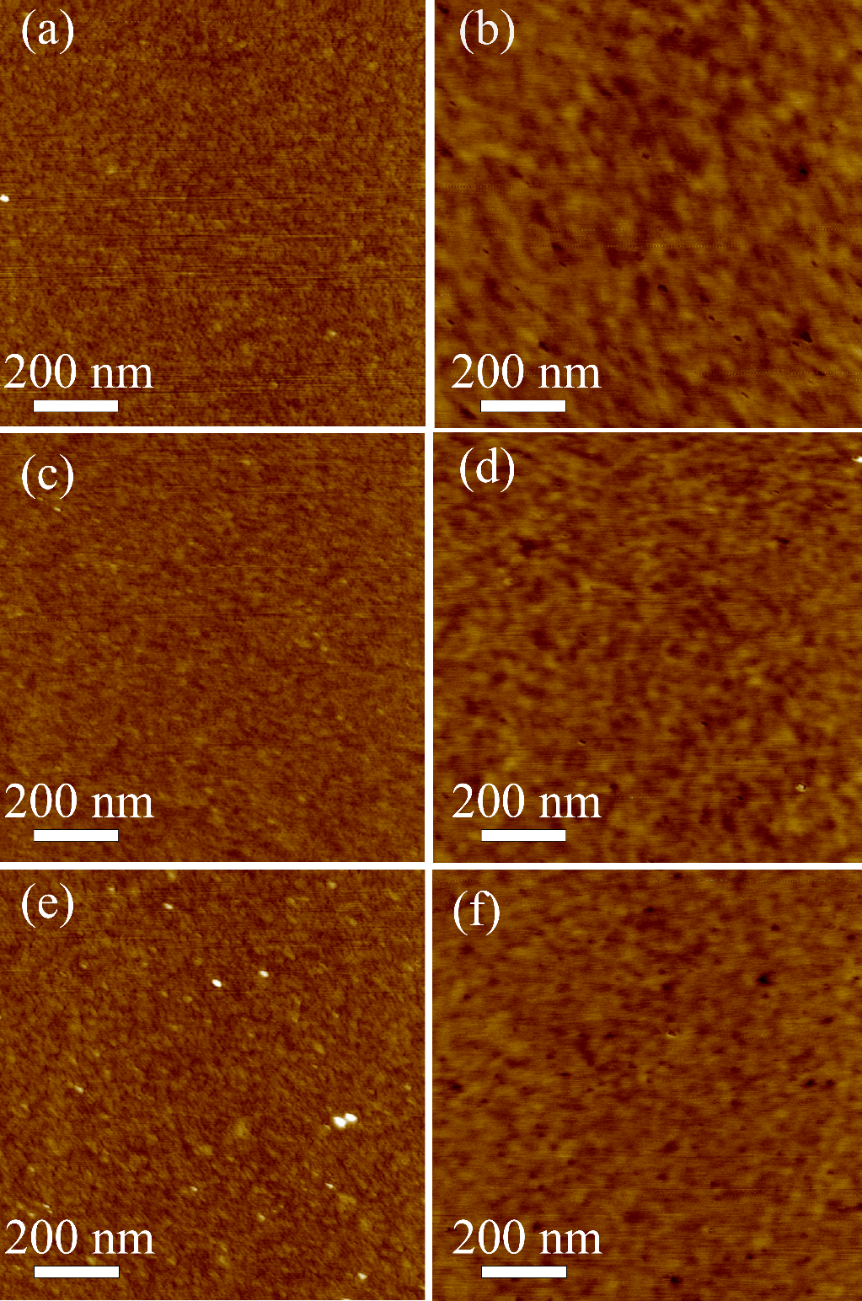
**

**F**igure S1. Higher magnification AFM topography images (1 µm^2^) recorded for: (a) 1% APTES‎, (b) 1% APTES_PEG/0.6 M ‎K_2_SO_4_/60°C‎, (c) 2% APTES‎, (d) 2%APTES_PEG/0.6 M K_2_SO_4_/60°C‎, (e) 4% APTES ‎and (f) 4% APTES_PEG/0.6 M K_2_SO_4_/60°C‎‎ (OD600nm = 0.48). All z-scales were equal to 5 nm. The higher magnification images clearly show the variation in topography between the APTES SAMs (left) and the APTES + PEG polymer layers (right).
